# Large cell neuroendocrine carcinoma (LCNEC) of the urinary bladder: a case report

**DOI:** 10.1186/1746-1596-8-19

**Published:** 2013-02-04

**Authors:** Cristina Colarossi, Piero Pino, Dario Giuffrida, Eleonora Aiello, Rosario Costanzo, Daniela Martinetti, Lorenzo Memeo

**Affiliations:** 1 Pathology Unit, Department of Experimental Oncology, Mediterranean Institute of Oncology, Via Penninazzo 7, Viagrande, (CT), Italy; 2 Urological Surgery Unit, Department of Experimental Oncology, Mediterranean Institute of Oncology, Via Penninazzo 7, Viagrande, (CT), Italy; 3 Oncology Unit, Department of Experimental Oncology, Mediterranean Institute of Oncology, Via Penninazzo 7, Viagrande, (CT), Italy; 4 IOM Ricerca Srl, Via Penninazzo 11, Viagrande, (CT), Italy

## Abstract

Neuroendocrine carcinoma of the urinary bladder is a rare entity, accounting less then 1% of urinary bladder malignancies. The vast majority of the neuroendocrine carcinoma of the urinary bladder is represented by small cell neuroendocrine carcinoma while just few cases of large cell neuroendocrine carcinoma (LCNEC) have been reported. In this cases report we describe a rare case of primary bladder LCNEC.

**Virtual Slides:**

The virtual slide(s) for this article can be found here: http://www.diagnosticpathology.diagnomx.eu/vs/2474700528951562.

## Background

Neuroendocrine carcinoma of the urinary bladder is a rare entity, accounting less than 1% of urinary bladder malignancies [1]. The vast majority of the neuroendocrine carcinomas of the urinary bladder is represented by small cell neuroendocrine carcinoma while just few cases of large cell neuroendocrine carcinoma (LCNEC) have been reported [1-9].

LCNEC was first described by Travis [10] in the lung but cases of LCNEC were reported in other organs like uterus, thymus, stomach, bile duct, larynx, parotid gland, prostate, kidney, and cervix [11,12].

In this cases report we describe a rare cases of primary bladder LCNEC.

## Case presentation

**Figure 1:**
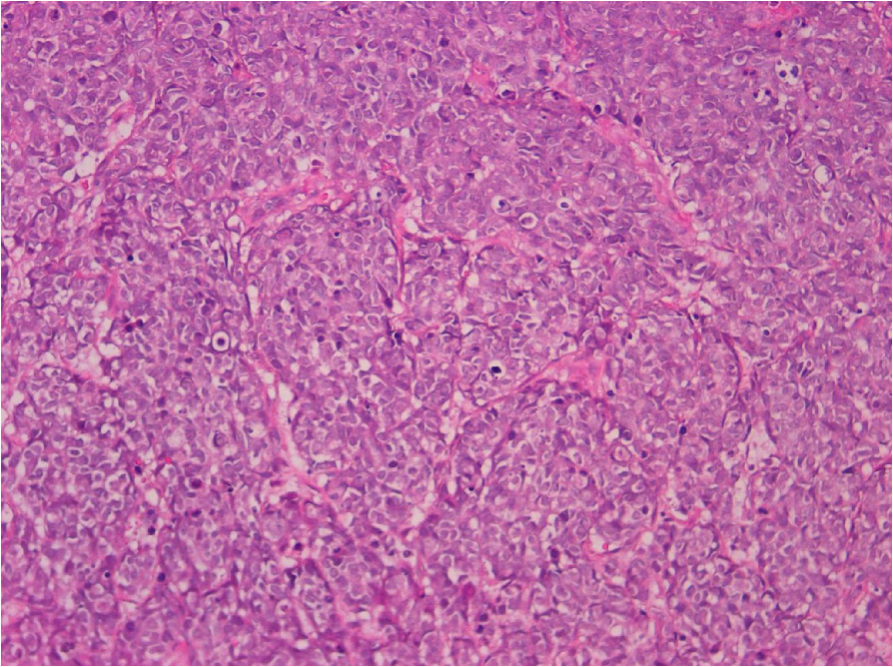
Neoplastic cells showing a sheet-like growth with trabecular and rosette-like patterns (H&E 20x).

A 53 years old woman presented with asymptomatic hematuria in September 2011. Ultrasound studies revealed a 4 cm mass in the posterior wall of the urinary bladder. TC scan confirmed the presence of the lesion and demonstrated that both ureteral opening were involved by the tumor. Mesenteric lymph nodes were evident. The tumor was partially resected transurethrally. Microscopically, the tumor was composed of large pleomorphic cells with moderate amount of cytoplasm and coarse nuclear chromatin, organized in trabecular and rosette-like patterns and showed high mitotic rate (Figure 1).

**Figure 2:**
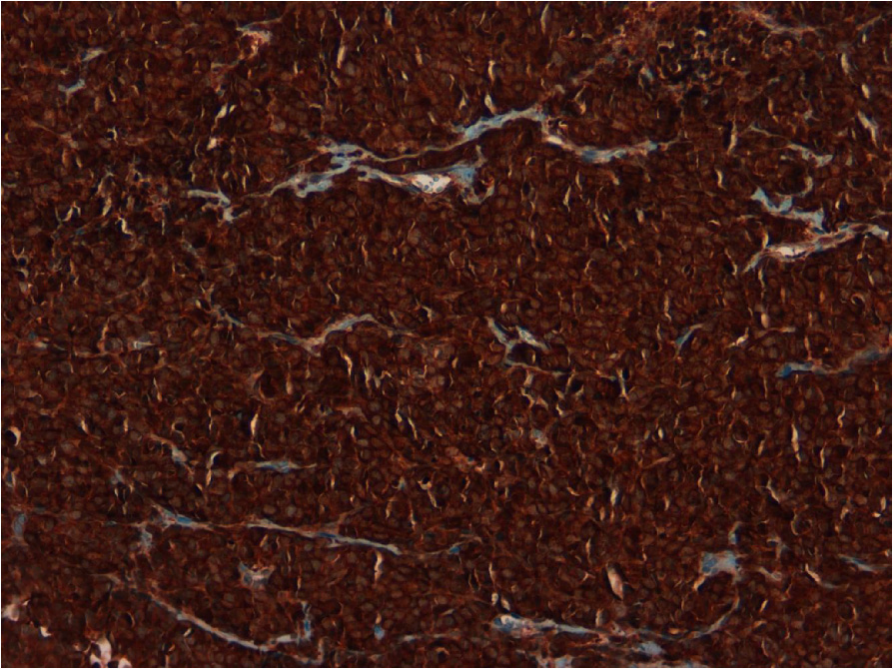
Tumor cells showed a diffuse and strong positivity for NSE (20x).

**Figure 3:**
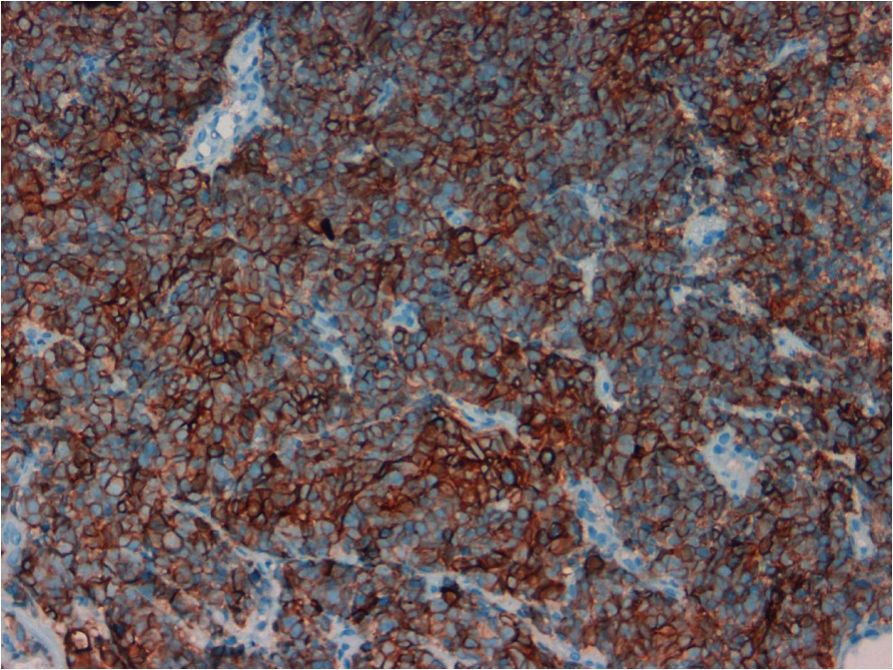
Tumor cells showed diffuse positivity for CD56 (20x).

**Figure 4:**
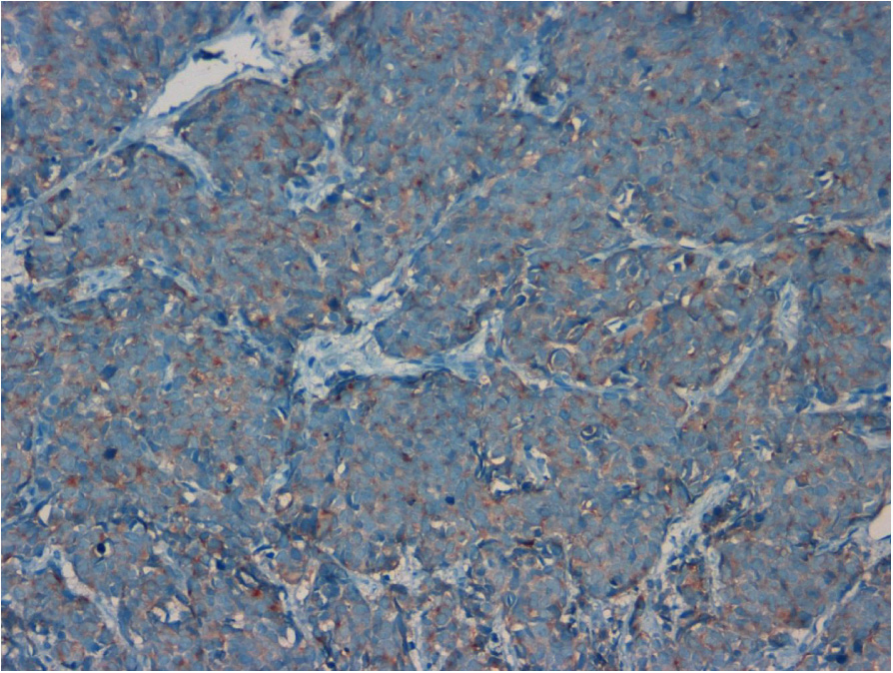
Tumor cells showed diffuse positivity for synaptophysin (20x).

**Figure 5:**
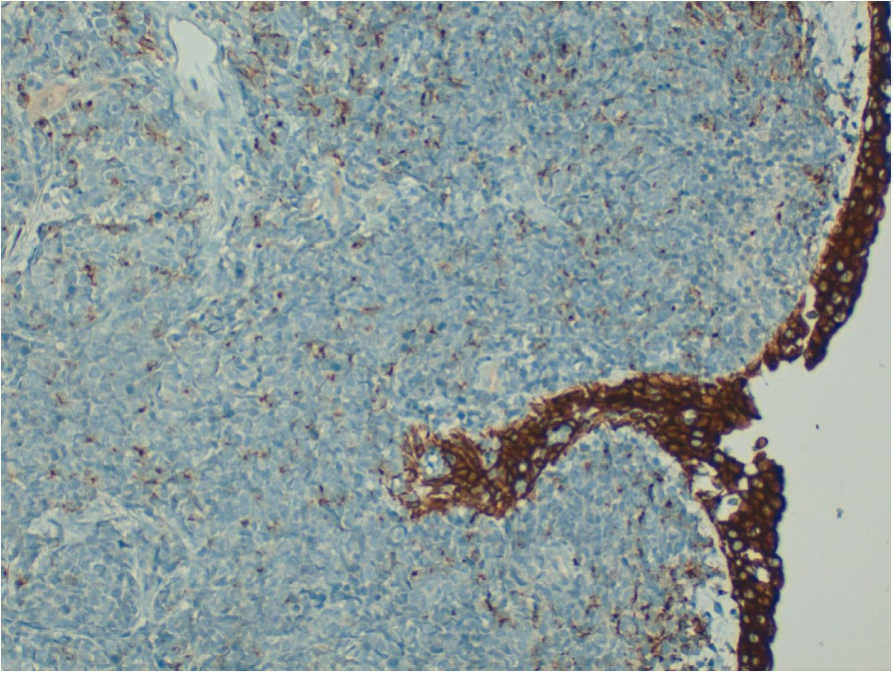
Tumor cells showed only focal positivity for AE1/AE3 in contrast with the diffuse positivity of the urothelium (20x).

**Figure 6:**
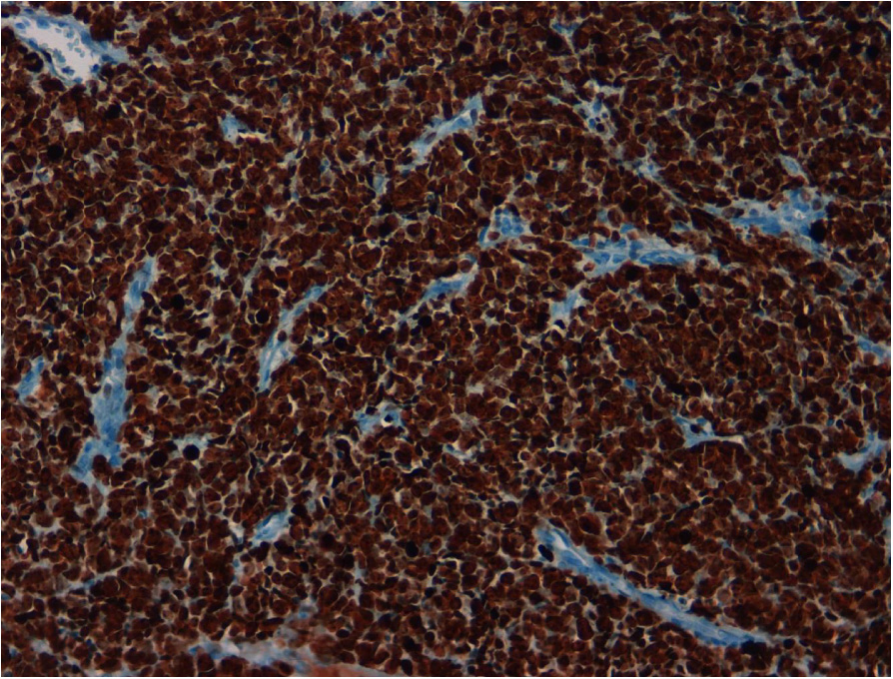
Ki-67 expression in virtually all neoplastic cells (20x).

Immunohistochemical analysis demonstrated that tumor cells were diffusely positive for NSE (Figure 2), CD56 (Figure 3) and synaptophysin (Figure 4), focally positive for chromogranin and pan-cytokeratin (Figure 5) and negative for high molecular weight cytokeratin and TTF-1. The proliferation index, evaluated with Ki-67 was >95% (Figure 6). Tumor extension to the suburothelial connective was seen as well as lymphovascular invasion. The patient was diagnosed with primary large cell neuroendocrine carcinoma (LCNEC) of the urinary bladder. Since we found in the literature [13] a case of large cell neuroendocrine carcinoma of the lung harboring EGFR mutation responding to Gefinitib, EGFR status was evaluated by direct sequencing, resulting wild type.

The patient was then treated with etoposide and cisplatin for 4 cycles. Reduction of the tumor and of the mesenteric lymph nodes size were seen at the end of the 4th cycle in December 2011. In January 2012 the patient underwent cystectomy with histero-annessectomy and lymphadenectomy in a different institution. At microscopic examination the diagnosis of LNEC was confirmed but area of conventional urothelial carcinoma were also seen associated with large amount of necrosis. The tumor showed infiltration of the perivesical tissue (pT3b) and metastasis were present in 5 right hypogastric lymph nodes (pN2) and in 2 common iliac lymph nodes (M1).

Following surgery, patient underwent a 5th cycle of chemotherapy with etoposide and cisplatin and she died on March 2012, seven months after the diagnosis.

## Conclusions

Neuroendocrine tumors of the urinary bladder are relatively rare and include carcinoids, large cell neuroendocrine carcinomas and small cell neuroendocrine carcinomas, the latter being by far the most common. LCNEC was first described in the lung by Travis [10], as well as the criteria for its diagnosis. These include the presence of polygonal large cells with a low nuclear/cytoplasmic ratio, coarse nuclear chromatin and evident nucleoli, high mitotic ratio (>10 mitoses/ 10 HPF) and immunohistochemical or ultrastructural evidence of neuroendocrine differentiation.

Only few cases of LCNEC are present in the literature and most of them showing mixed histology, including the present case. In our case, areas of LCNEC and urothelial carcinoma were evident while in other reported cases were described areas of LCNEC mixed with a squamous cell or adenocarcinoma component. This is in line with the hypothesis that neuroendocrine cells originate from stem cells suggesting a common clonal origin of the neuroendocrine, urothelial, squamous or adenocarcinoma component [14].

Mixed histology is also seen in other rare primary bladder tumors such as adult rhabdomyosarcoma [15] or giant cell tumor [16].

Interestingly, around 30% of cases of small cell carcinoma or the urinary bladder are positive for TTF-1 [2] while no evidence of expression of TTF-1 was found in LCNEC in the literature and in the present case.

In addition, to our knowledge, this is the first case in the literature of primary LCNEC of the bladder with known status of EGFR.

In our case, as well as in most of the described cases, tumor showed a very rapid local growth in few months, and this might be one of the important clinical manifestations of this rare tumor.

Recognition of this rare entity could be significant considering its poor outcome and differential diagnosis considerations. The differential diagnosis of primary LCNEC includes metastatic LCNEC, extension from poorly differentiated prostatic carcinomas, high grade urothelial carcinoma, small cell neuroendocrine carcinoma and lymphoma.

## Consent

Written informed consent was obtained from the patient for publication of this case report and any accompanying images. A copy of the written consent is available for review by the Editor-in-Chief of this journal.

## Competing interests

The authors declare that they have no conflict of interest.

## Authors’ contributions

CC conceived of the study, participated in its design and drafted the manuscript. PP participated in the design of the study. DG participated in the design of the study. EA carried out the IHC study. RC carried out the molecular studies. DM carried out the molecular studies. LM participated in the design of the study and drafted the manuscript. All authors read and approved the final manuscript.
